# Supplementary material for: Preparation and Application of Organic-Inorganic Nanocomposite Materials in Stretched Organic Thin Film Transistors
Source: Polymers (Basel). 2020 May 5;12(5):1058. doi: 10.3390/polym12051058 (PMC7284877; doi:10.3390/polym12051058)
Supplement: Supplementary file 1 [file polymers-12-01058-s001.pdf]

Supporting Information

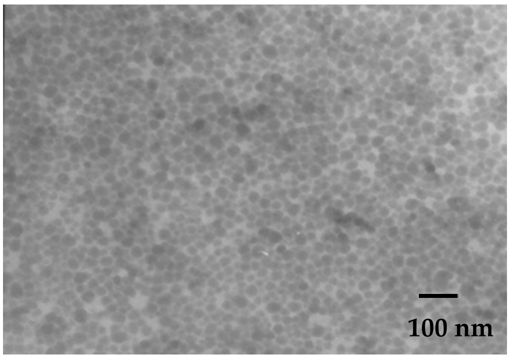

Figure S1. The TEM image of inorganic TiO_2-SiO_2 nanoparticles.

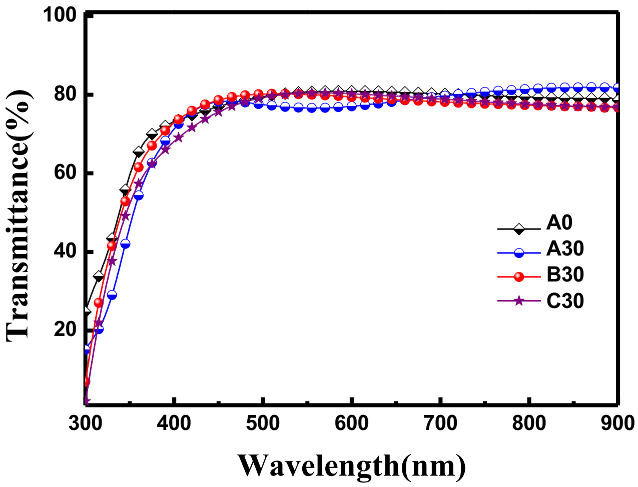

Figure S2. The optical transmittance of A0, A30, B30, and C30 films as the dielectric layer of OTFTs device.

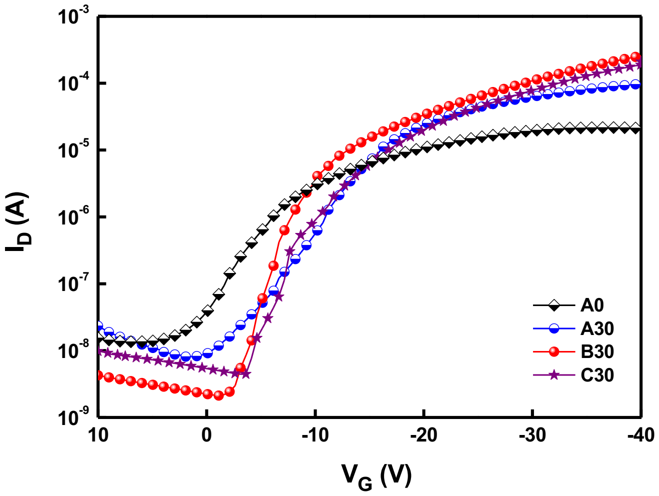

Figure S3. The transfer curves of OTFTs prepared by different dielectric layer materials, A0, A30, B30, and C30.

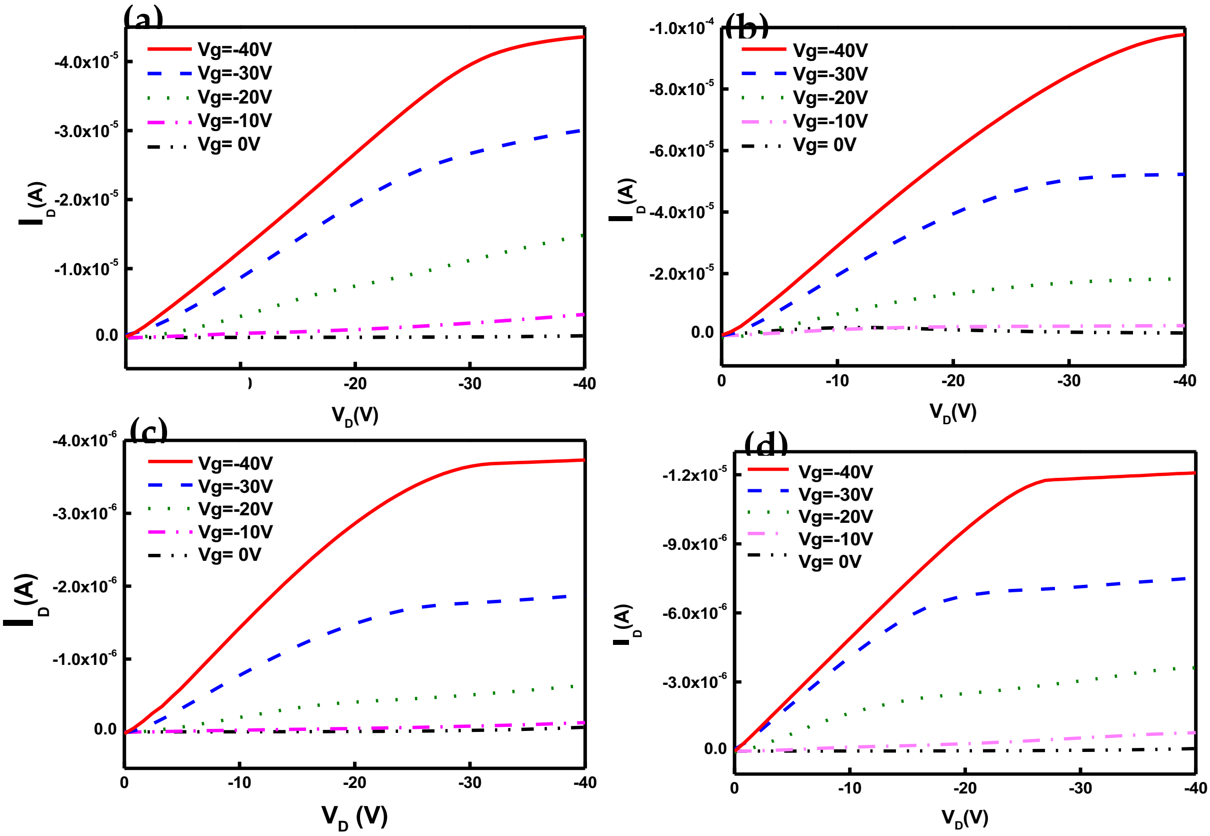

Figure S4. The output characteristics of the OTFTs prepared by different dielectric layer materials: (a) A0, (b) A30, (c) B30, and (d) C30.
